# Clinical trials in drug development: the Brazilian experience

**DOI:** 10.1186/1753-6561-8-S4-O2

**Published:** 2014-10-01

**Authors:** Odorico de Morais

**Affiliations:** 1 Universidade Federal do Ceará, Fortaleza, Brazil

## 

Clinical research in Brazil today can be seen in three distinct areas. In the first, still acting shy and with few resources, the research is funded through government agencies that seek the understanding of some diseases or peculiar conditions in the country. These include neglected diseases still endemic in Brazil that do not interest large international industries. In the second area, funded with abundant resources coming from international pharmaceutical industries, research studies are carried out to evaluate the efficacy and safety of drugs in multicenter trials. The third area, new in the country, is the clinical research of bioavailability and bioequivalence of drugs that emerged due to the need for assessing the quality of generic drugs, which was funded in part with public funds and in part with funds from national pharmaceutical industries. Although there are criticisms to all three, they have contributed to boosting clinical research in the country. At the moment, it is necessary to use a policy to guide their future directions and coordinate the actions of groups that have academic competence and are already working traditionally in the sector, avoiding the opportunism that often takes place. It is always important to keep in mind that clinical research is not done only by dilettantism or on behalf of scientific knowledge. This research involves humans and therefore it should be conducted in compliance with ethical principles of autonomy, beneficence, non-maleficence, justice and equity, concerning the participation of the research subject and the social relevance of their results. It should also be considered that the research carried out in Brazil by major international pharmaceutical conglomerates had the merit of introducing the country to the international circuit of multicenter clinical research, to emphasize the quality of our scientists in academia and to introduce new research protocols. However, neither the design of these experiment nor the elaboration of protocols and even the management of the experiments were in charge of our research groups. When they are not the responsibility of the industry itself, they are outsourced to international CROs (Contract Research Organizations), leaving the Brazilian researcher only in an operational role. In another scenario we find domestic industries that are still groping in drug research and development. However, some have begun to realize the impossibility of surviving in the market doing just similar and generic drugs. Therefore, it is necessary that our clinical research is prepared to support the national pharmaceutical industry that invests in the development of new drugs, and do so with the same competence displayed in the quality control of generic medications. Furthermore, it is of critical importance that clinical research is also prepared to assist public and private institutions in obtaining information regarding the diseases prevalent in the population.

